# 

**Published:** 1853-02

**Authors:** 


					﻿NEW BOOKS AND COMMUNICATIONS.
Our correspondents whose papers have been for some time on hand
must exercise a little patience. We shall probably be able to give their
esteemed favors a place in our next number. We have received many
new books which we shall notice at an early day. Among these are the
5th volume of the Transactions of the American Medical Association,
and the new edition of Kirkes’ and Paget’s Physiology.
Receipts of Medical Journal for January, 1853.
Dr. J. H. Matthews, Canton, Ohio,	-	-	-85 00
M. L. Embree, Rockhouse Prarie, Mo.,	-	-	10 00
---- Shepherd, Newbern,’ Ind.,	-	.	-	5	00
W. G. Rhodes, Rocky Hill, Ky.,	-	-	-	5	00
B. F. Smith, Warrensburg, Miss.,	-	-	-	5	00
W. M. Burks. Center lAO.^ Ky.,	-	.	.	20	00
-----Stapp, Lewisport, Ky., -	-	-	-	10 00
R. B. Shelby, Henderson, Ky.,	-	-	-	10	00
G.	Holts, Canton,Ohio.	-	-	-	-	10	00
Drs. McKee & Smith, Sharon, Miss.,	-	-	-	5	00
Dr. J. W. Sanders Carrollville, Miss.,	-	-	-	5	00
P. Taylor, Chalk Level, Ky. -	-	-	-	2	50
J.	G. Bridges, Clinton College, Tenn., -	-	-	5	00
R. A. New, Rodney, Miss., -	-	-	-	5	00
A. J. Merich, Freedom, Ind., -	-	-	-	5	00
W. S. Cooper, New Albany, Ind.,	-	-	-	5	00
P. H. Buchanan, Cane Creek, Tenn.,	-	-	-	5 00
K.	Routh, La Grange, Texas,	-	-	-	-	5	00
H.	Mosely, Halifax, Miss.,	-	-	-	-	3	00
G. M. Lee, Sugar Grove, Ky.,	-	-	-	-	5	00
J. Swain, Ballardsville, Ky.,	-	-	-	-	5	00
J. J. Stovall, Danville, Ala.,	-	-	-	-	5	00
Drs. Pollock & Savage, Germantown, Ky., -	-	-	5	00
Dr. J. H. McKay, Big Spring, Ky.,	-	-	-	-	5	00
A. T. Porter, White Plains, Ala.,	-	-	5 00
J. McBride, Smith’s Fork, Tenn.,	-	-	-	5 00
E. L. Minor, Lethopolis, Ohio, -	-	-	-	5 00
The Westers Journal of Medicine and Surgery is published monthly by
the undersigned, at the corner of Main and Fifth streets, Louisville, at $5 per
annum, payable in advance. Each number contains 96 pages, making two vol-
umes in the year of more than 550 pages each.
Contributions to its pages by the Physicians of the Valley of the Mississippi
addressed to the Editors, are respectfully solicited.
Letters on business, to be addressed,postage paid, to the publishers.
PRENTICE &, HENDERSON
				

